# Supplementary material for: Multi-Omics and network-based exploration of potential molecular pathways in heart failure relevant to left bundle branch pacing response heterogeneity: Immune remodeling, hub gene identification, and drug repurposing hypotheses
Source: PLoS One. 2026 May 11;21(5):e0348601. doi: 10.1371/journal.pone.0348601 (PMC13160336; doi:10.1371/journal.pone.0348601)
Supplement: Attachment — Submitted filename: Response to Reviewers.docx [file pone.0348601.s002.docx]

Dear Dr. Redoy Ranjan and Reviewers,

We are grateful to the Academic Editor and the four reviewers for their constructive and insightful feedback on our manuscript (PONE-D-25-56864). Their comments have significantly strengthened the work by highlighting areas for improved clarity, rigor, and transparency. We have carefully addressed each point raised, resulting in substantial revisions to the manuscript. Key changes include reframing the study as hypothesis-generating based on HF transcriptomics as proxies for LBBP mechanisms, expanding methodological details with mathematical formulations and parameters, depositing all source code in a public repository for reproducibility, and enhancing the limitations section to acknowledge data constraints. These revisions are highlighted in the tracked-changes version of the manuscript. Below, we provide a point-by-point response to the reviewers' comments.

**Reviewer #1:**

Comment: In Multi-Omics and Network-Based Dissection of Left Bundle Branch Pacing in Heart Failure: Immune Remodeling, Hub Gene Identification, and Drug Repurposing Opportunities“ Sun et al. re-analyse three rather old gene expression data sets (published between 2006-2016) in order to gain (molecular) insights into heterogeneous clinical responses to left bundle branch pacing (LBBP). Note that LBBP was introduced in 2017! So it is not clear to me how diffentially expressed genes are detected given that the three data sets - on top – have rather different study designs. The rest of the paper is built on top of this rather unclear basis and the authors continue with method descriptions that are rather generic with very little transparency with regard to what has actually been done.

Response: We thank Reviewer #1 for this critical observation regarding the temporal mismatch between the datasets and LBBP's introduction, as well as the need for greater methodological transparency. We fully agree that direct inference of LBBP-specific mechanisms from pre-2017 HF datasets would be inappropriate due to potential confounders and design differences. To address this, we have reframed the study's scope throughout the manuscript (e.g., revised title, abstract, introduction, and discussion) to position the analysis as exploratory, using conserved HF molecular features as proxies for potential LBBP response heterogeneity. We have added a dedicated limitations subsection explicitly acknowledging this constraint and calling for validation in LBBP-specific cohorts. For methodological transparency, we have expanded all sections with specific algorithms, equations, parameters, and software versions.

**Reviewer #2:**

Comment: How the preprocessing carried out and other classification, PCA and Matrix details could be explained with mathematical mode. which tool supported the authors to conclude their findings. Gene sequence and other medicine terms may not be verified by my review.

Response: We appreciate Reviewer #2's suggestion to enhance mathematical and tool-based explanations. We have added explicit mathematical formulations and details for preprocessing (e.g., PCA variance as eigenvalues / sum(eigenvalues), ComBat parametric adjustment), differential expression (e.g., empirical Bayes model: expression ~ group + error), functional enrichment (hypergeometric distribution equation), immune profiling (ν-SVR linear system), WGCNA (soft-thresholding and adjacency equations), hub genes (kWithin summation), and drug prediction (Kolmogorov-Smirnov statistics). All analyses were performed in R (version 4.3.2) with specified packages (e.g., limma, sva, clusterProfiler, WGCNA, PharmacoGx).

**Reviewer #3:**

Comment: I preface this review by reminding the editors and author that I am not a Cardiologist. I am not, outside of the nature of the datasets used. My review will be related to the machine learning methodology, reproducibility of the results and statistics. Reproducibility: I would request that the authors submit, in some fashion, the source code for the project. Without it, reproducibility would be extremely challenging. Data Quality: Although the GEO Datasets are publicly available as stated, they are Heart Failure datasets, not specific datasets for the modality LBBP. The datasets do not contain individuals, that I could see, underwent LBBP. This creates a circular logic problem for the model and the outputs are heart failure biology, not LBBP specific. Without the full dataset to evaluate and the source code, the unified transcriptomic resource created is for heart failure. Prehaps review of the full dataset will reveal some specific LBBP data but current data and design do not support author claims. To complete a comprehensive review for the manuscript both the raw and processed data and source code would need to be submitted.

Response: We thank Reviewer #3 for emphasizing reproducibility and data specificity. To ensure full reproducibility, we have deposited all source code (R scripts for preprocessing, DESeq2/limma, WGCNA, CIBERSORT, etc.), raw/processed matrices, and intermediate results in compressed attachment. Regarding data quality and circular logic, we agree that the datasets reflect general HF biology rather than LBBP-specific effects. We have reframed the manuscript to clarify this, interpreting findings as hypothesis-generating proxies requiring LBBP cohort validation. No LBBP-specific samples were in the datasets, as noted.

**Reviewer #4:**

Comment: The research paper “Multi-Omics and Network-Based Dissection of Left Bundle Branch Pacing in Heart Failure” presents a comprehensive integrative transcriptomic analysis aimed at characterizing the molecular landscape associated with left bundle branch pacing (LBBP). While the study appears promising, the manuscript would benefit from a more detailed explanation of the statistical models and computational algorithms used for differential expression analysis, functional enrichment, immune infiltration profiling, weighted gene co-expression network analysis (WGCNA), hub gene identification, and drug–pathway prediction.

Response: We thank Reviewer #4 for recognizing the study's promise and for requesting detailed explanations. We have expanded the methods with specific statistical models and algorithms: differential expression (limma eBayes model), enrichment (hypergeometric tests), immune profiling (CIBERSORT ν-SVR), WGCNA (adjacency/TOM matrices), hub genes (kWithin connectivity), and drug prediction (Kolmogorov-Smirnov scores).

We believe these revisions fully address the concerns and enhance the manuscript's suitability for publication. We look forward to your feedback.

Sincerely,

Mingfeng Jin (Corresponding Author)

On behalf of all authors
